# Neuronal overexpression of hTDP-43 in *Caenorhabditis elegans* mimics the cellular pathology commonly observed in TDP-43 proteinopathies

**DOI:** 10.17912/micropub.biology.000767

**Published:** 2023-04-19

**Authors:** Mandy Koopman, Lale Güngördü, Renée I. Seinstra, Wytse Hogewerf, Ellen A. A. Nollen

**Affiliations:** 1 European Research Institute for the Biology of Ageing, University of Groningen, University Medical Centre Groningen, The Netherlands

## Abstract

Inclusions consisting of transactive response DNA-binding protein 43 (TDP-43) are a characteristic feature of amyotrophic lateral sclerosis (ALS). *Caenorhabditis elegans* has been instrumental in studying the underlying mechanisms of TDP-43 pathology. Here, we extend the possibilities of previous studies by examining a *C. elegans* model expressing human wild-type *TDP-43* ( *hTDP-43* ) pan-neuronally. We show that disease-related (hyper)phosphorylation and cytosolic localisation of hTDP-43 are present in hTDP-43 worms and that these features can be enhanced by adjusting the environmental temperature.

**Figure 1. Pan-neuronal expression of hTDP-43 in C. elegans results in molecular phenotypes that resemble those seen in TDP-43-proteinopathies:**
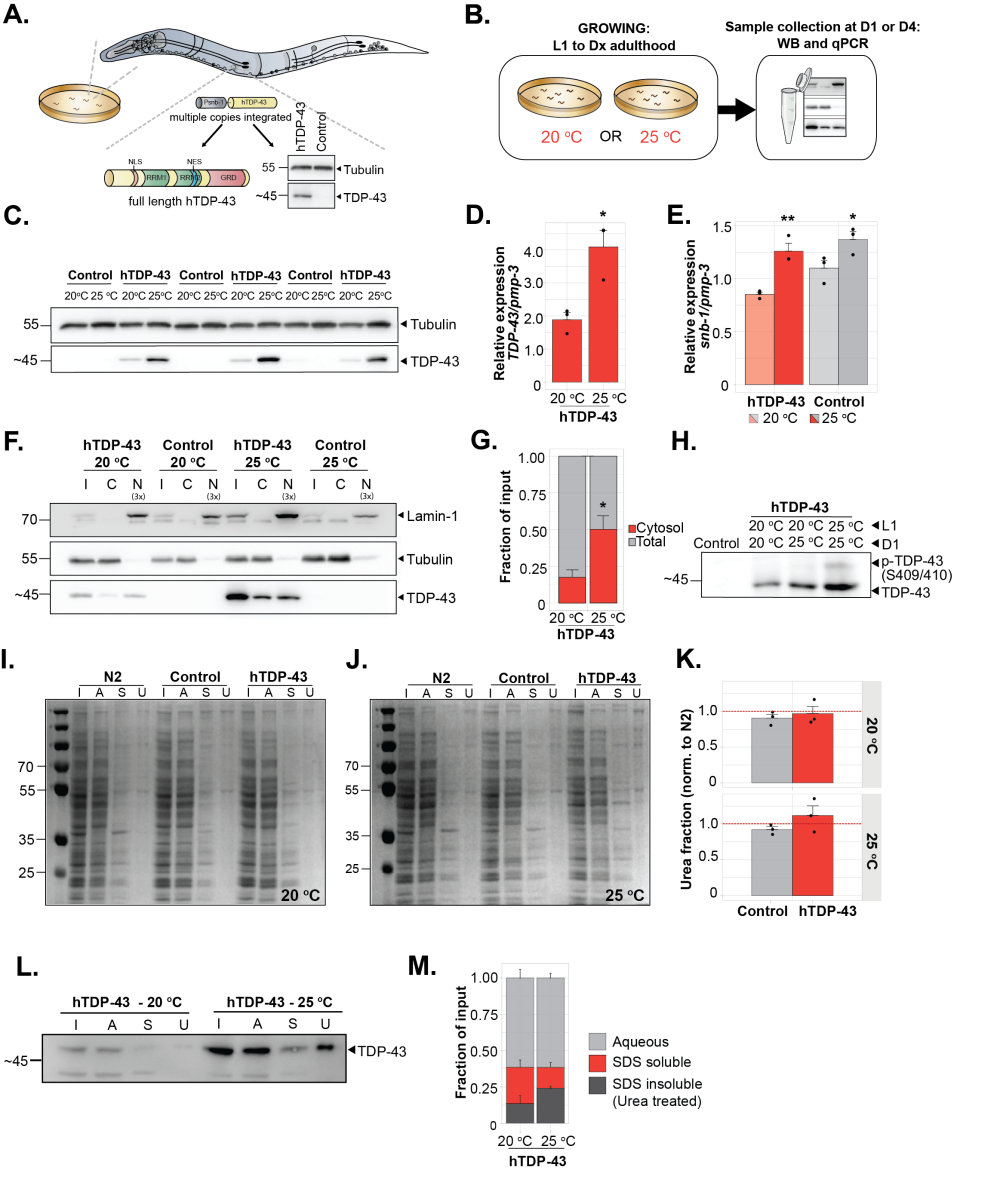
**A)** A schematic showing the *C. elegans* model used in this study. hTDP-43 is expressed under the synaptobrevin-1 ( *snb-1)* promoter to drive expression pan-neuronally ( *snb-1p::hTDP-43/3’long UTR + mtl-2p::GFP)* . Control worms used throughout the panel: *mtl-2p::GFP.* **B)** A schematic showing the experimental outline. For each experiment worms were consistently raised at either 20 °C or 25 °C starting from larval stage 1 (L1) for 72 (~D1) or 148 hours (~D4), unless stated differently. **C)** Western blot of hTDP-43 levels at different growth temperatures (adulthood D1), *n* = 3. **D)** Relative expression of hTDP-43, normalized by *pmp-3* , at different temperatures as measured with a quantitative PCR (adulthood D1), *n* = 3, two-tailed unpaired Student’s t test: p = 0.0154. **E)** Relative RNA expression of *snb-1* , normalized by *pmp-3* , at different temperatures as measured with a quantitative PCR (adulthood D1), *n=3,* two-tailed unpaired Student’s t test for hTDP-43: p = 0.0061, for the control strain: p = 0.043. **F)** Western blot of subcellular fractionation of hTDP-43. LMN-1 (nuclear marker) and α-tubulin (cytosolic marker) were used as controls (adulthood D1). The nuclear fraction was concentrated 3 times for each condition to get a clear signal for TDP-43. I= input, C = cytosolic, N = nuclear. **G)** Quantification of the fraction hTDP-43 in the cytosolic compartment (see **F** ) compared to the input signal. *n=3,* two-tailed unpaired Student’s t test: p = 0.0348. **H)** Western blot of hTDP-43 and p-TDP-43 (S409/S410). Worms were either grown at 20 °C or 25 °C from L1 until D4, or worms were grown until D1 at 20 °C and then switched to 25 °C (20 °C / 25 °C) until D4, *n* = 1. **I)** Coomassie staining of proteome-wide solubility in control and hTDP-43 worms at 20 °C (adulthood D1). I = input, A = aqueous soluble, S = SDS soluble, U = urea soluble. **J)** Coomassie staining of proteome-wide solubility in control and hTDP-43 worms at 25 °C (adulthood D1). I = input, A = aqueous soluble, S = SDS soluble, U = urea soluble. **K)** Quantification of coomassie staining. The urea-to-input ratio was normalized by N2 worms, *n = 3,* two-tailed unpaired Student’s t test for 20 °C and 25 °C: p = n.s. **L)** Western blot of solubility of hTDP-43. Samples from I and J were used to blot for hTDP-43 in the different fractions. **M)** Quantification of the fraction hTDP-43 in the SDS-soluble and urea-soluble fractions (see **L** ) compared to the input signal. *n=3,* two-way ANOVA (temperature, interaction: n.s., fraction: p<0.001) with post-hoc Sidak’s. Error bars represent S.E.M. *: p ≤ 0.05, **: p ≤ 0.01, ***: p ≤ 0.001.

## Description

Transactive response DNA binding-protein 43 (TDP-43) is a highly conserved RNA/DNA-binding protein with a pivotal role in RNA metabolism and homeostasis (Tziortzouda et al., 2021) . Cytoplasmic inclusions consisting of TDP-43 are specific pathological features of TDP-43 proteinopathies, including frontotemporal dementia (FTD) and amyotrophic lateral sclerosis (ALS) (Neumann et al., 2006; Arai et al., 2006) . Several studies have shown that *Caenorhabditis elegans* is a suitable model to phenocopy ALS and FTD. Especially the overexpression of mutated forms of TDP-43 in *C. elegans* has been shown to govern cellular toxicity and to capture several molecular hallmarks seen in TDP-43 (Ash et al., 2010; Vaccaro et al., 2012; Zhang et al., 2011; Zhang et al., 2012; Liachko et al., 2010) . Characteristic post-translation modifications, insolubility and cytosolic localization of mutant TDP-43 have all been observed in these *C. elegans* models. However, these molecular features are either absent or less pronounced in *C. elegans* models expressing wild-type TDP-43 (Ash et al., 2010; Liachko et al., 2010; Vaccaro et al., 2012) . Here we show that the characteristic cellular hallmarks of TDP-43-proteinopathies are displayed in *C. elegans* model overexpressing wild-type *TDP-43* when the environmental temperature is raised.

To broadly investigate the proteotoxicity of wild-type TDP-43 at the molecular level, we used a *C. elegans* model expressing human *TDP-43* in a pan-neuronal fashion under the promotor of *snb-1* (hereafter referred to as hTDP-43 worms) (Ash et al., 2010) . Since hTDP-43 worms also express a fluorescent gut marker ( *Pmtl-2::GFP* ), worms expressing the same marker were used as controls (Ash et al., 2010) ( **Figure 1A** ). The effect of temperature on TDP-43 aggregation has previously been established in *C. elegans* . Switching the environmental temperature of hTDP-43 worms from 20 °C to 25 °C increases the aggregation propensity of TDP-43 and results in the accumulation of the protein in aggregates (Zhang et al., 2011) . We made use of the notion that the molecular hallmarks of TDP-43 can be altered by changing its thermal environment and assessed all molecular phenotypes in hTDP-43 worms grown at 20 °C or 25 °C ( **Figure 1B** ).

First, we established the effect of temperature on the levels of hTDP-43. Raising the growth temperature of hTDP-43 worms from 20 °C to 25 °C substantially increased hTDP-43 protein levels ( **Figure 1C** ). The increased hTDP-43 levels were, however, mirrored at the transcriptional level for both *hTDP-43* ( **Figure 1D** ) and its promotor, *snb-1* ( **Figure 1E** ). Therefore, increased protein levels of hTDP-43 can at least partially be attributed to the temperature-sensitivity of the *snb-1* promotor.

Given the notion that cytosolic localisation, phosphorylation, and insolubility are all characteristic hallmarks of TDP-43-proteinopathies (Arai et al., 2006; Neumann et al., 2006; Winton et al., 2008; Neumann et al., 2009; Wegorzewska et al., 2009; Barmada et al., 2010; Da Cruz et al., 2011; Hasegawa et al., 2011; Halliday et al., 2012; Arnold et al., 2013; Liachko et al., 2014; Sugai et al., 2019) , we subsequently investigated whether hTDP-43 worms show any of these molecular features. To investigate the relative nuclear-to-cytosolic localisation of hTDP-43, which is normally concentrated in the nucleus, we applied fractionation methods (Mata-Cabana et al., 2018) . In line with previous studies, we found the largest fraction of hTDP-43 to be present in the nucleus (Ash et al., 2010; Vaccaro et al., 2012) ( **Figure 1F, G** ). However, when the temperature was raised to 25 °C, hTDP-43 localisation shifted towards the cytosolic compartment. Furthermore, exposing worms to an environmental temperature of 25 °C increased hTDP-43 phosphorylation of residues S409/S410 ( **Figure 1H** ), a hTDP-43 feature that was nearly absent at 20 °C. The relative insolubility of hTDP-43, on the other hand, was not significantly altered by different growth temperatures ( **Figure 1I -M** ). Noteworthy, while hTDP-43 turned more insoluble at 25 °C in absolute measurements ( **Figure 1L,M** ) the total proteome solubility was not affected when compared to control conditions ( **Figure 1I -K** ). Since TDP-43 has previously been reported to mainly aggregate in the cytosol, the extent of observed TDP-43 aggregation may be underestimated by our whole-cell extract fractionation procedure.

Taking everything together, our results show that neuronal expression of wild-type hTDP-43 in *C. elegans* captures the characteristic molecular hallmarks of TDP-43 proteinopathies especially at higher growth temperatures.

## Methods

**Strains and maintenance**

Standard conditions were used for *C. elegans* propagation at 20 °C (Brenner, 1974) . Animals were age-synchronized by hypochlorite bleaching and subsequently allowed to hatch overnight in M9 buffer at 20 °C. For experiments age synchronized L1s were cultured for 72h at either 20 °C or 25 °C on NGM plates seeded with OP50 before being tested, unless stated differently.

**Western blotting/SDS-PAGE**

Worms were collected in PBS supplemented with Complete Protease Inhibitor (Roche; #11697498001) and then sonicated for 15 cycles: 30 sec on, 15 sec off. Worm debris was spun down and the protein concentration was determined using the PierceTM BCA Protein Assay Kit (Thermo Fischer; #23225) according to manufacturer’s protocol. 20-30µg protein was dissolved in 5x laemmli buffer and boiled at 95 °C for 10 minutes. Subsequently, samples were loaded on a 12% Tris-Glycine acrylamide gel. Gels were transferred to a 0.2µm nitrocellulose membrane (Bio-Rad; #1620112) or an activated 0.2µm PVDF membrane (Millipore;#ICEQ00010). Membranes were blocked with 5% milk in PBS-T (0.1%) for 1 hour. For protein detection the following antibodies were used: anti-tdp-43 (abnova; # H00023435-M01); 1:1000 in 5% milk, anti-tubulin (Sigma Aldrich; # T6074); 1:10.000 in 5% milk, anti-phospho tdp-43 (S409/410) (Cosmobio; #TIP-PTD-M01); 1:500 in 3% BSA and anti-lmn-1 (Novus Biologicals; #38530002); 1:1000 in 5% milk. Membranes were incubated with primary antibodies overnight at 4°C. Membranes were incubated with the secondary antibody (1:10.000) for 1 hour at room temperature (goat anti- mouse IgG (H+L)-HRP conjugate (Bio-Rad; #170-6516) or goat anti- Rabbit IgG (H+L)-HRP conjugate (Bio-Rad; #170-6515)). Antibody binding was visualized using Amersham ECL Prime Western Blotting Detection Reagent (GE healthcare; #RPN2236) and imaged with the ImageQuant LAS4000 Imaging unit (GE Healthcare).

**Fractionation**

D1 worms were resuspended in hypotonic buffer (15 mM HEPES KOH pH 7.6, 10 mM KCl, 5 mM MgCl _2_ , 0.1 mM EDTA, 350 mM Sucrose, 1 mM DTT, supplemented with protease inhibitors cocktail (Complete, Roche). Worms were subsequently homogenized using a pellet pestle by applying 5-7 strokes of 1 min followed by 1 min rest on ice between the strokes. Next, worm debris was removed by centrifugation at 500 *g* for 5 min at 4 °C. The supernatant was collected as used as input of the cell fractionation (25 μl was collected in a separate tube for immunoblotting). Nuclei were separated by spinning the supernatant at 4000 *g* for 5 min at 4 °C. The supernatant (containing the cytosolic fraction) was removed and subsequently centrifuged at 17.000 *g* for 30 min at 4 °C. to remove further contaminants. The nuclear pellet was washed two times with hypotonic buffer (and centrifuged at 500 *g* for 5 min at 4 °C) before resuspending it in hypertonic buffer (15 mM HEPES KOH pH 7.6, 400 mM KCl, 5 mM MgCl _2_ , 0.1 mM EDTA, 0.1% Tween 20, 10% Glycerol, 1 mM DTT, supplemented with protease inhibitors cocktail (Complete, Roche)) – in 1/3 of the volume of the cytosolic fraction (enriching the nuclear fraction 3 times). Finally, the nuclear fraction was treated with 25 U/μl Benzonase (Millipore) for 30 min at 4 °C (Mata-Cabana et al., 2018) .

**Solubility**

Worms (D1) were resuspended in lysis buffer (50 mM Tris/HCl pH 7.4, 100 mM NaCl, complete protease inhibitor cocktail (Roche Diagnostics)), homogenized by 5-7 20s cycles with a FastPrep-24 (MP Biomedicals) at 4 m/s, and clarified by low-speed centrifugation (2000 *g)* . Protein content was measured and equalized to 400 μg using the PierceTM BCA Protein Assay Kit (Thermo Fischer; #23225) according to manufacturer’s protocol. A whole cell extract fraction (input, I) was taken and subsequently the rest of sample was pelleted by high-speed centrifugation (20.000 *g* for 25 minutes at 4 °C). The supernatant was set apart (aqueous phase, A) and the pellet was resuspended in lysis buffer and pelleted again as an extra washing step (the supernatant was discarded). Then, the pellet was resuspended in the same volume of lysis buffer with 1% SDS and placed at heating block (600 rpm, RT) for 30 minutes. When the pellet was completely dissolved, the proteins were pelleted by high-speed centrifugation (20.000 *g* for 25 minutes at 4 °C) to yield a supernatant containing the SDS-soluble fraction. A small fraction was set apart (SDS-soluble, S) and the pellet was resuspended and pelleted again as an extra washing step (the supernatant was discarded). Finally, the pellet with SDS-insoluble proteins was solubilized in the same volume of urea buffer (8M urea, 2% v/v SDS, 50 mM DTT, 50 mM Tris/HCl, pH 7.4) first 1 hour at 60 °C and subsequently overnight at RT in a Thermomixer R (Eppendorf) at 1200 rpm (yielding the Urea-soluble fraction, U). The I, A, S-fractions were mixed with sample buffer and boiled for 10 minutes at 95 °C. The same volumes of all fractions were separated using SDS-PAGE and visualized with coomassie staining.

**Quantitative PCR**

To assess the gene expression levels, total RNA from Day 1 adult *C.* *elegans* was isolated using TRizol (Invitrogen; #15596018) according to manufacturer’s protocol. The RNA quality and concentration were assessed with a NanoDrop 2000 spectrophotometer (Thermo Scientific). From 1 µg total RNA, cDNA was made using the RevertAid H Minus First Strand cDNA Synthesis kit (Thermo Scientific; #K1632), using random hexamer primers. The quantitative real-time PCR was performed with a Roche LightCycler 480 Instrument II (Roche diagnostics), using 2 µl of 1:10 cDNA dilution. SYBR green dye (Bio-Rad; #172-5125) was used for detection of cDNA amplification. Relative transcript levels were quantitated using a standard curve of pooled cDNA samples. Expression levels were normalized to endogenous reference gene *pmp-3* .

## Reagents

**Table 1:** Strains used

**Table:** 

**Strain**	**Description**	**Genotype**	**Remark**
N2	Bristol wild isolate	Wildtype	
OW1601	CL6049 6x backcrossed with N2 . Named: hTDP-43 worms.	*dvIs62* [ *snb-1 p::hTDP-43/3’long UTR + mtl-2p::GFP* ]X	Provided by Chris Link
OW1603	CL2122 6x backcrossed with N2 . Named: control worms.	*dvIs15* [( *pPD30.38) unc-54 (vector) + (pCL26)mtl-2p::GFP* ]	Provided by Chris Link

**Table 2:** Primers

**Table:** 

**Gene**	**Forward Primer (qPCR)**		**Reverse Primer (qPCR)**	
*hTDP-43*	GGTGGTGGGATGAACTTTGG		TGCTGGCTGGCTAACATGC	
*pmp-3*	CACTCATCTCTATGACGACGTTTC		CACCGTCGAGAAGCTGTAGA	
*snb-1*	CAGGTTGATGAAGTCGTCGG		ATTGTGAAGCACCTTCCTGG	
